# Supplementary material for: Cryo-EM structures of RAD51 assembled on nucleosomes containing a DSB site
Source: Nature. 2024 Mar 20;628(8006):212–20. doi: 10.1038/s41586-024-07196-4 (PMC10990931; doi:10.1038/s41586-024-07196-4)
Supplement: Supplementary file 1 — Supplementary Figs 1–5 – the replicated and uncropped gels. [file 41586_2024_7196_MOESM1_ESM.pdf]

nature portfolio

https://doi.org/10.1038/s41586-024-07196-4

Supplementary information

Cryo-EM structures of RAD51 assembled on nucleosomes containing a DSB site

In the format provided by the authors and unedited

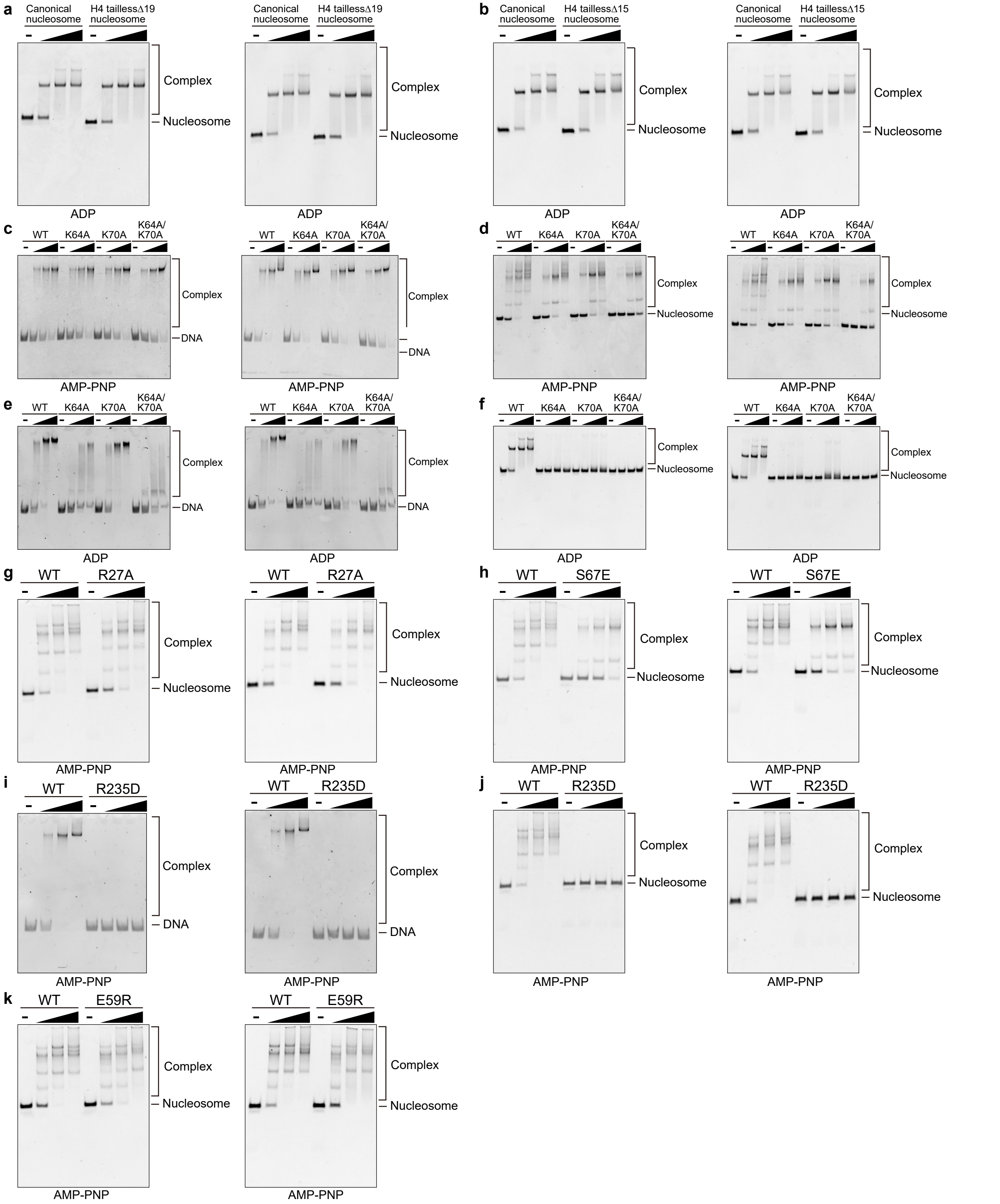

Supplementary Fig. 1: Replicated gel images of electrophoretic mobility shift assays of RAD51 and nucleosomes.

a, Replicated electrophoretic mobility shift assays of RAD51 with the canonical nucleosome and H4 taillessΔ19 nucleosome in the presence of ADP.

b, Replicated electrophoretic mobility shift assays of RAD51 with the canonical nucleosome and H4 taillessΔ15 nucleosome in the presence of ADP.

c, Replicated electrophoretic mobility shift assay of RAD51, RAD51(K64A), RAD51(K70A) and RAD51(K64A/K70A) with the DNA in the presence of AMP-PNP.

d, Replicated electrophoretic mobility shift assay of RAD51, RAD51(K64A), RAD51(K70A) and RAD51(K64A/K70A) with the nucleosome in the presence of AMP-PNP.

e, Replicated electrophoretic mobility shift assay of RAD51, RAD51(K64A), RAD51(K70A) and RAD51(K64A/K70A) with the DNA in the presence of ADP.

f, Replicated electrophoretic mobility shift assay of RAD51, RAD51(K64A), RAD51(K70A) and RAD51(K64A/K70A) with the nucleosome in the presence of ADP.

g, Replicated electrophoretic mobility shift assays of RAD51 and RAD51(R27A) with the nucleosome in the presence of AMP-PNP.

h, Replicated electrophoretic mobility shift assays of RAD51 and RAD51(S67E) with the nucleosome in the presence of AMP-PNP.

i, Replicated electrophoretic mobility shift assays of RAD51 and RAD51(R235D) with the DNA in the presence of AMP-PNP.

j, Replicated electrophoretic mobility shift assays of RAD51 and RAD51(R235D) with the nucleosome in the presence of AMP-PNP.

k, Replicated electrophoretic mobility shift assays of RAD51 and RAD51(E59R) with the nucleosome in the presence of AMP-PNP.

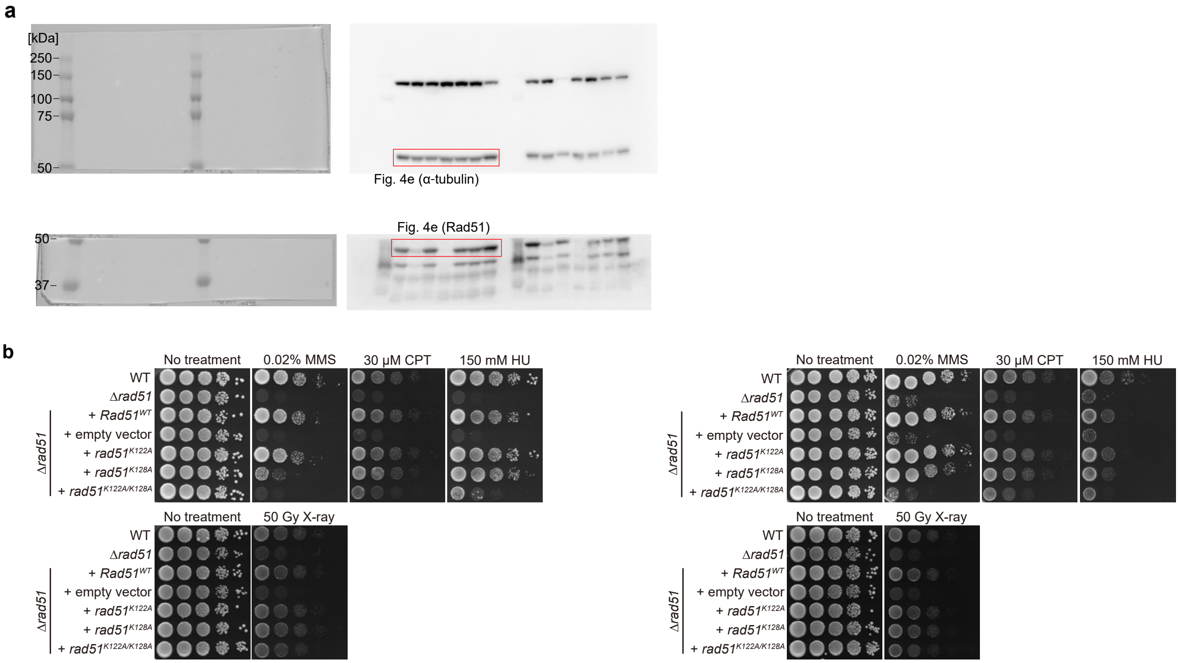

Supplementary Fig. 2: Uncropped membranes and replicated spot assays.

a, Uncropped membranes of western blot detecting the Rad51 and α-tubulin.

b, Replicated spot assays for assessing DNA of yeast cells producing *S. cerevisiae* Rad51 mutants. Spot assays were performed using independent strains of each mutant.

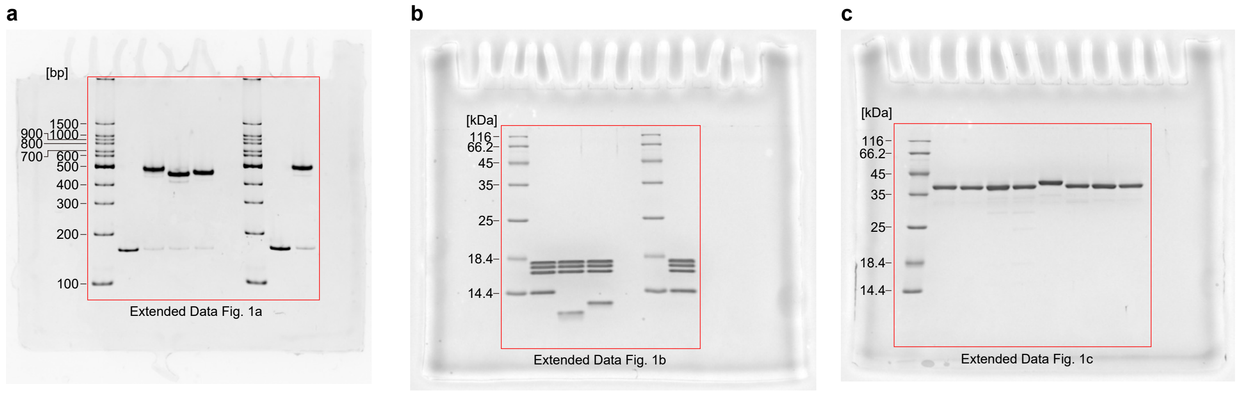

Supplementary Fig. 3: Uncropped gel images for preparation of the nucleosome and RAD51.
a, Uncropped gel images for Extended Data Fig. 1a.
b, Uncropped gel images for Extended Data Fig. 1b.
c, Uncropped gel images for Extended Data Fig. 1c.

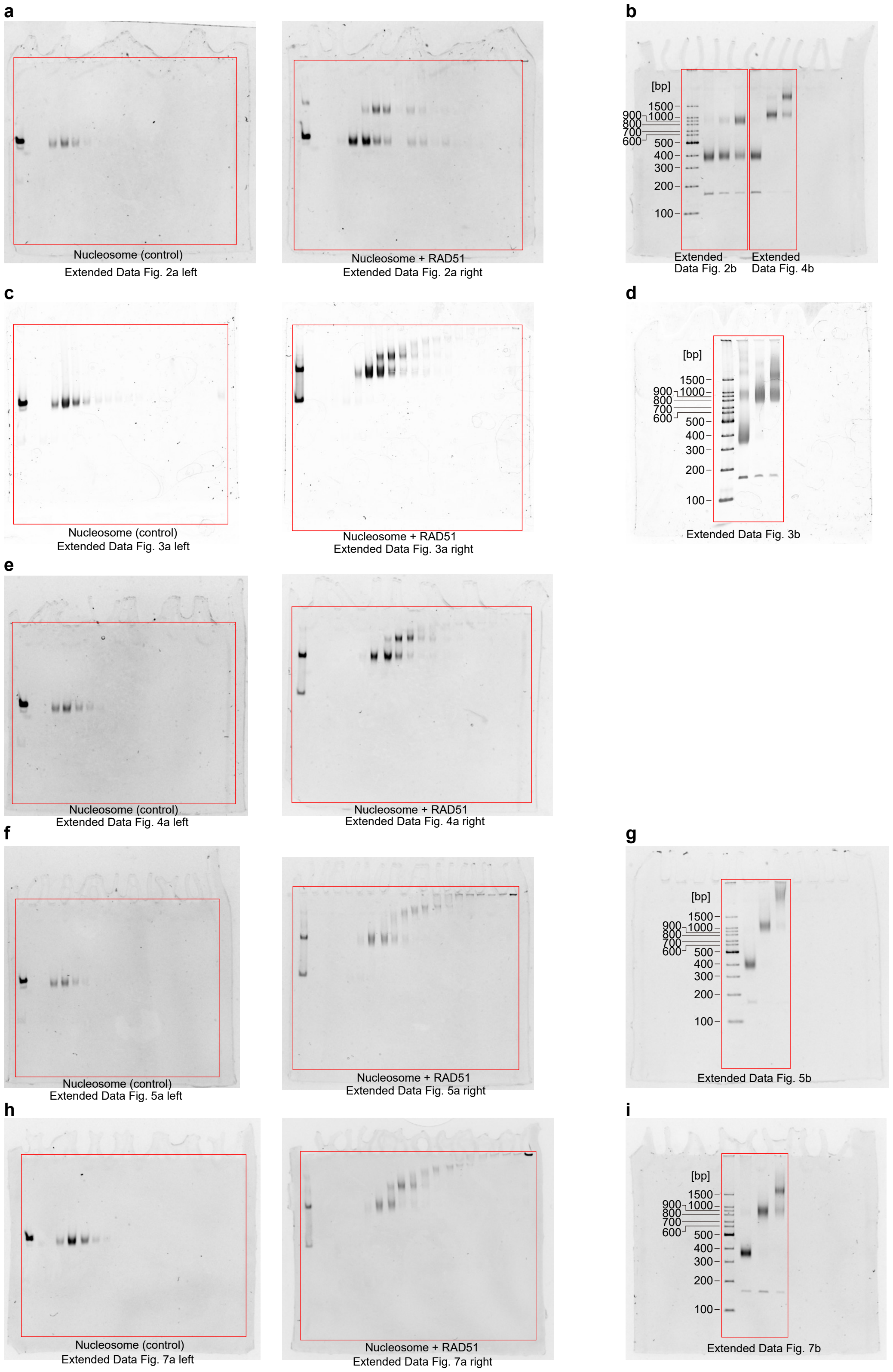

Supplementary Fig. 4: Uncropped gel images for preparation of the RAD51-nucleosome complexes.
a, Uncropped gel images for Extended Data Fig. 2a.
b, Uncropped gel images for Extended Data Fig. 2b and 4b.
c, Uncropped gel images for Extended Data Fig. 3a.
d, Uncropped gel images for Extended Data Fig. 3b.
e, Uncropped gel images for Extended Data Fig. 4a.
f, Uncropped gel images for Extended Data Fig. 5a.
g, Uncropped gel images for Extended Data Fig. 5b.
h, Uncropped gel images for Extended Data Fig. 7a.
i, Uncropped gel images for Extended Data Fig. 7b.

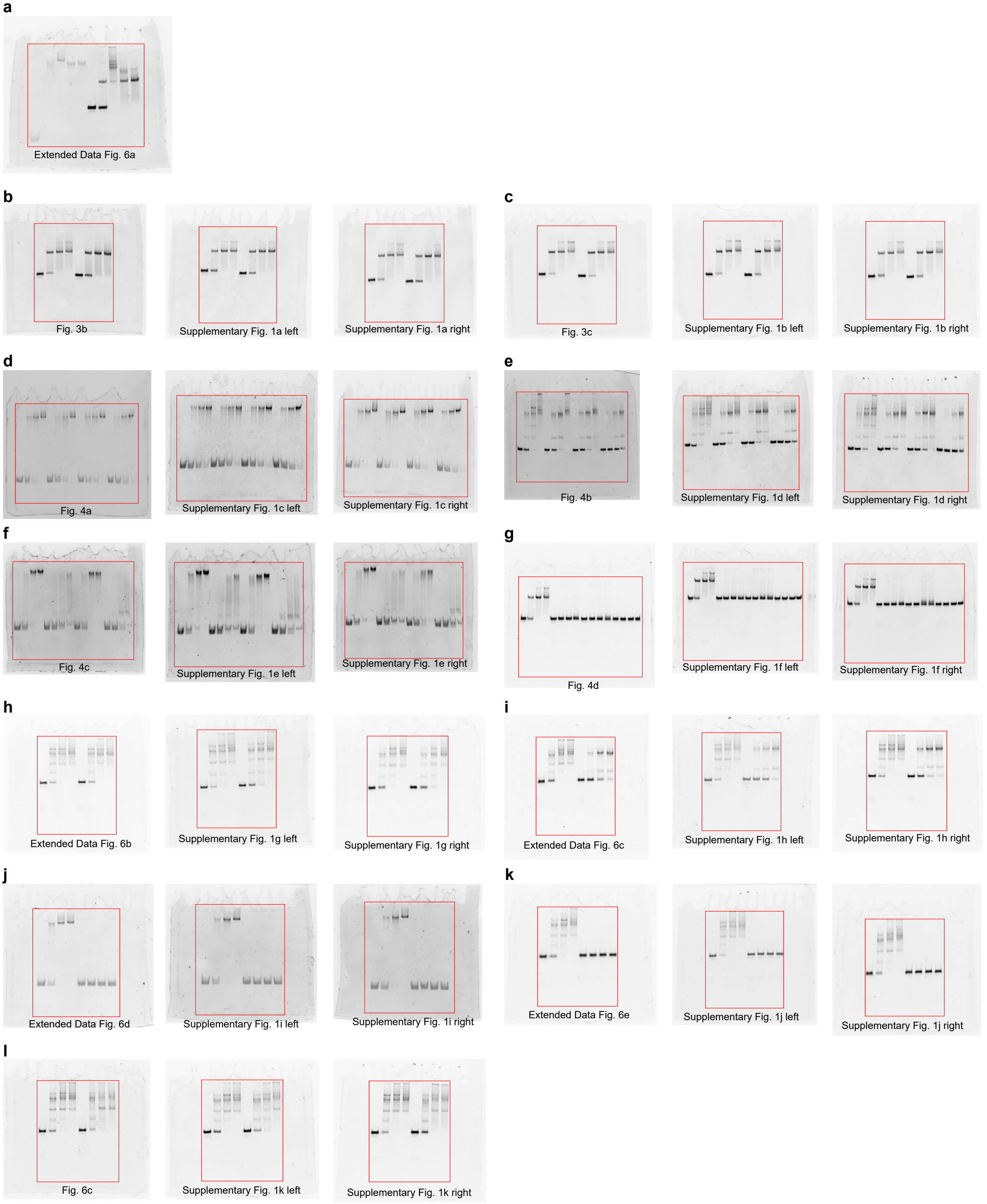

Supplementary Fig. 5: Uncropped gel images for electrophoretic mobility shift assays.
a, Uncropped gel images for Extended Data Fig. 6a.
b, Uncropped gel images for Fig. 3b and Supplementary Fig. 1a.
c, Uncropped gel images for Fig. 3c and Supplementary Fig. 1b.
d, Uncropped gel images for Fig. 4a and Supplementary Fig. 1c.
e, Uncropped gel images for Fig. 4b and Supplementary Fig. 1d.
f, Uncropped gel images for Fig. 4c and Supplementary Fig. 1e.
g, Uncropped gel images for Fig. 4d and Supplementary Fig. 1f.
h, Uncropped gel images for Extended Data Fig. 6b and Supplementary Fig. 1g.
i, Uncropped gel images for Extended Data Fig. 6c and Supplementary Fig. 1h.
j, Uncropped gel images for Extended Data Fig. 6d and Supplementary Fig. 1i.
k, Uncropped gel images for Extended Data Fig. 6e and Supplementary Fig. 1j.
l, Uncropped gel images for Fig. 6c and Supplementary Fig. 1k.
