# Supplementary material for: Cryo-EM structures of RAD51 assembled on nucleosomes containing a DSB site
Source: Nature. 2024 Mar 20;628(8006):212–20. doi: 10.1038/s41586-024-07196-4 (PMC10990931; doi:10.1038/s41586-024-07196-4)
Supplement: Supplementary file 2 — Reporting Summary [file 41586_2024_7196_MOESM2_ESM.pdf]

nature portfolio

Corresponding author(s): Hitoshi Kurumizaka

Last updated by author(s): Feb 5, 2024

Reporting Summary

Nature Portfolio wishes to improve the reproducibility of the work that we publish. This form provides structure for consistency and transparency in reporting. For further information on Nature Portfolio policies, see our Editorial Policies and the Editorial Policy Checklist.

Statistics

For all statistical analyses, confirm that the following items are present in the figure legend, table legend, main text, or Methods section.

n/a	Confirmed	
☐	☒	The exact sample size (*n*) for each experimental group/condition, given as a discrete number and unit of measurement
☐	☒	A statement on whether measurements were taken from distinct samples or whether the same sample was measured repeatedly
☐	☒	The statistical test(s) used AND whether they are one- or two-sided<br>*Only common tests should be described solely by name; describe more complex techniques in the Methods section.*
☐	☒	A description of all covariates tested
☐	☒	A description of any assumptions or corrections, such as tests of normality and adjustment for multiple comparisons
☐	☒	A full description of the statistical parameters including central tendency (e.g. means) or other basic estimates (e.g. regression coefficient) AND variation (e.g. standard deviation) or associated estimates of uncertainty (e.g. confidence intervals)
☐	☒	For null hypothesis testing, the test statistic (e.g. *F*, *t*, *r*) with confidence intervals, effect sizes, degrees of freedom and *P* value noted<br>*Give P values as exact values whenever suitable.*
☒	☐	For Bayesian analysis, information on the choice of priors and Markov chain Monte Carlo settings
☒	☐	For hierarchical and complex designs, identification of the appropriate level for tests and full reporting of outcomes
☒	☐	Estimates of effect sizes (e.g. Cohen's *d*, Pearson's *r*), indicating how they were calculated

Our web collection on statistics for biologists contains articles on many of the points above.

Software and code

Policy information about availability of computer code

Data collection	EPU 3.1
Data analysis	Relion 4.0-beta-2, MotionCor2 1.4.0, ctffind-4.1.14, UCSF ChimeraX 1.2, 1.3, 1.4, 1.5 and 1.6, ISOLDE 1.3 and 1.4, Coot 0.9.8.1, PHENIX 1.20, ImageJ 1.53e and 1.53f51, ImageQuant TL ver.8.1, Topaz, R 4.3.2, Python 3.11.7.

For manuscripts utilizing custom algorithms or software that are central to the research but not yet described in published literature, software must be made available to editors and reviewers. We strongly encourage code deposition in a community repository (e.g. GitHub). See the Nature Portfolio guidelines for submitting code & software for further information.

Data

Policy information about availability of data

All manuscripts must include a data availability statement. This statement should provide the following information, where applicable:

- Accession codes, unique identifiers, or web links for publicly available datasets
- A description of any restrictions on data availability
- For clinical datasets or third party data, please ensure that the statement adheres to our policy

The cryo-EM structures and the atomic models of the RAD51-nucleosome complexes have been deposited in the Electron Microscopy Data Bank (EMDB) and the Protein Data Bank (PDB), respectively. The accession codes are as follows: EMD-38228 and PDB ID 8XBT for the cryo-EM structure of the octameric RAD51 ring bound to the nucleosome with the linker DNA binding, EMD-36442 and PDB ID 8JND for the cryo-EM structure of the nonameric RAD51 ring bound to the

nucleosome with the linker DNA binding, EMD-38229 and PDB ID 8XBU for the cryo-EM structure of the decameric RAD51 ring bound to the nucleosome with the linker DNA binding, EMD-36443 and PDB ID 8JNE for the cryo-EM structure of the decameric RAD51 ring bound to the nucleosome without the linker DNA binding, EMD-36444 and PDB ID 8JNF for the cryo-EM structure of the RAD51 filament bound to the nucleosome, EMD-38230 and PDB ID 8XBV for the cryo-EM structure of RAD51 L1 and L2 loops bound to the linker DNA with the sticky end of the nucleosome, EMD-38231 and PDB ID 8XBW for the cryo-EM structure of the RAD51 N-terminal lobe domain bound to the histone H4 tail of the nucleosome, EMD-38232 and PDB ID 8XBX for the cryo-EM structure of RAD51 L2 loop bound to the linker DNA with the blunt end of the nucleosome, EMD-38233 and PDB ID 8XBY for the cryo-EM structure of RAD51 L1 and L2 loops bound to the linker DNA with the blunt end of the nucleosome. Uncropped images are shown in Supplementary Figs. 2-5. Source data for the quantification are provided with this paper.

Research involving human participants, their data, or biological material

Policy information about studies with human participants or human data. See also policy information about sex, gender (identity/presentation), and sexual orientation and race, ethnicity and racism.

Reporting on sex and gender	N/A
Reporting on race, ethnicity, or other socially relevant groupings	N/A
Population characteristics	N/A
Recruitment	N/A
Ethics oversight	N/A

Note that full information on the approval of the study protocol must also be provided in the manuscript.

Field-specific reporting

Please select the one below that is the best fit for your research. If you are not sure, read the appropriate sections before making your selection.

☒ Life sciences ☐ Behavioural & social sciences ☐ Ecological, evolutionary & environmental sciences

For a reference copy of the document with all sections, see nature.com/documents/nr-reporting-summary-flat.pdf

Life sciences study design

All studies must disclose on these points even when the disclosure is negative.

Sample size	Sample sizes in all assays were three independent experiments. Sample size calculation was not conducted, as three independent experiments are sufficient for the biological statical analysis.
Data exclusions	No data was excluded from the analysis.
Replication	Three independent experiments were performed in the electrophoretic mobility shift assays, the western blots and the DNA damage sensitivity assays with S. cerevisiae. All replicated experiments were performed successfully. Cryo-EM analysis was conducted once because the final map already represented the average of a large number of images.
Randomization	S. cerevisiae strains were randomly allocated to the DNA damage sensitivity assays. There were no other experiments which required randomization.
Blinding	Since there was no subjective allocation in our investigation, blinding was irrelevant to this study

Reporting for specific materials, systems and methods

We require information from authors about some types of materials, experimental systems and methods used in many studies. Here, indicate whether each material, system or method listed is relevant to your study. If you are not sure if a list item applies to your research, read the appropriate section before selecting a response.

Materials & experimental systems

n/a	Involved in the study
☐	☒ Antibodies
☒	☐ Eukaryotic cell lines
☒	☐ Palaeontology and archaeology
☒	☐ Animals and other organisms
☒	☐ Clinical data
☒	☐ Dual use research of concern
☒	☐ Plants

Methods

n/a	Involved in the study
☒	☐ ChIP-seq
☒	☐ Flow cytometry
☒	☐ MRI-based neuroimaging

Antibodies

Antibodies used

Anti-S. cerevisiae Rad51 rabbit antibody (BioAcademia, cat #62-101) , HRP-conjugated anti-rabbit IgG (1:5,000; Merck; NA9340), and HRP-conjugated anti-tubulin alpha (Bio-Rad, cat #MCA77P) were used in the western blots.

Validation

Anti-S. cerevisiae Rad51 rabbit antibody (BioAcademia, cat #62-101)
Western blot of crude extract of S. cerevisiae was performed.https://www.bioacademia.co.jp/html/upload/save_image/0318174257_623446113b0d3.pdf

HRP-conjugated anti-tubulin alpha (Bio-Rad, cat #MCA77P)
western blot of HeLa whole cell lysate was performed,https://www.bio-rad-antibodies.com/monoclonal/yeast-tubulin-alpha-antibody-yl1-2-mca77.html?f=hrp
